# The frequent and clinically benign anomalies of chromosomes 7 and 20 in Shwachman-diamond syndrome may be subject to further clonal variations

**DOI:** 10.1186/s13039-021-00575-w

**Published:** 2021-11-24

**Authors:** Abdul Waheed Khan, Alyssa Kennedy, Elissa Furutani, Kasiani Myers, Annalisa Frattini, Francesco Acquati, Pamela Roccia, Giovanni Micheloni, Antonella Minelli, Giovanni Porta, Marco Cipolli, Simone Cesaro, Cesare Danesino, Francesco Pasquali, Akiko Shimamura, Roberto Valli

**Affiliations:** 1 Genetica Umana e Medica, Dipartimento di Medicina e Chirurgia, Università Dell’Insubria, Via J. H. Dunant, 5, 21100 Varese, Italy; 2 Dana Farber/Boston Children’s Cancer and Blood Disorders Center, Boston, MA USA; 3 Keros Therapeutics, Lexington, MA USA; 4 Cincinnati Children’s Hospital and Medical Center, Cincinnati, OH USA; 5 Istituto di Ricerca Genetica e Biomedica, CNR, Milano, Italy; 6 Dipartimento di Biotecnologie e Scienze della Vita, Università Dell’Insubria, Varese, Italy; 7 Genetica Medica, Fondazione IRCCS Policlinico S. Matteo and Università di Pavia, Pavia, Italy; 8 Centro Fibrosi Cistica, Azienda Ospedaliera Universitaria Integrata, Verona, Italy; 9 Oncoematologia Pediatrica, Azienda Ospedaliera Universitaria Integrata, Verona, Italy; 10 Centro di Medicina Genomica—Università dell’Insubria, Varese, Italy

**Keywords:** Bone marrow rescue, Chromosome anomalies, Karyotype instability, Shwachman-Diamond syndrome

## Abstract

**Background:**

An isochromosome of the long arm of chromosome 7, i(7)(q10), and an interstitial deletion of the long arm of chromosome 20, del(20)(q), are the most frequent anomalies in the bone marrow of patients with Shwachman-Diamond syndrome, which is caused in most cases by mutations of the *SBDS* gene. These clonal changes imply milder haematological symptoms and lower risk of myelodysplastic syndromes and acute myeloid leukaemia, thanks to already postulated rescue mechanisms.

**Results:**

Bone marrow from fourteen patients exhibiting either the i(7)(q10) or the del(20)(q) and coming from two large cohorts of patients, were subjected to chromosome analyses, Fluorescent In Situ Hybridization with informative probes and array-Comparative Genomic Hybridization. One patient with the i(7)(q10) showed a subsequent clonal rearrangement of the normal chromosome 7 across years. Four patients carrying the del(20)(q) evolved further different del(20)(q) independent clones, within a single bone marrow sample, or across sequential samples. One patient with the del(20)(q), developed a parallel different clone with a duplication of chromosome 3 long arm. Eight patients bore the del(20)(q) as the sole chromosomal abnormality. An overall overview of patients with the del(20)(q), also including cases already reported, confirmed that all the deletions were interstitial. The loss of material varied from 1.7 to 26.9 Mb and resulted in the loss of the *EIF6* gene in all patients.

**Conclusions:**

Although the i(7)(q) and the del(20)(q) clones are frequent and clinically benign in Shwachman Diamond-syndrome, in the present work we show that they may rearrange, may be lost and then reconstructed de novo*,* or may evolve with independent clones across years. These findings unravel a striking selective pressure exerted by *SBDS* deficiency driving to karyotype instability and to specific clonal abnormalities.

**Supplementary Information:**

The online version contains supplementary material available at 10.1186/s13039-021-00575-w.

## Background

Shwachman-Diamond syndrome (SDS) is an autosomal recessive disorder (Online Mendelian Inheritance in Man #260400) characterized by exocrine pancreatic insufficiency, bone marrow failure, peripheral cytopenias and an increased risk of developing aplastic anaemia, myelodysplastic syndrome (MDS) and acute myeloid leukaemia (AML). The patients may also exhibit several additional signs. SDS is caused by mutations of the *SBDS* gene in at least 90% of cases. [1] SBDS protein has been associated with different functions, mainly playing a role in ribosome biogenesis or RNA processing [2] and, as other genes belonging to this group, it is highly conserved in all eukaryotes [1, 3]. Recently, it has been well characterized that SBDS protein play a pivotal role in the early ribosome biogenesis by releasing the anti-association factor EIF6 from the pre-60S ribosome subunit thus allowing the formation of the mature 80S ribosome [4]. However, SDS is genetically heterogeneous. Biallelic mutations of two other genes involved in ribosome biogenesis may cause SDS, or an SDS-like condition: *DNAJC21*,[5, 6] and *EFL1* [7]. An SDS-like phenotype may be also caused by monoallelic mutations of the gene *SRP54*, which produces a protein that is a key member of the cotranslation protein-targeting pathway [8]. Therefore, SDS may be considered a ribosomopathy.

Clonal chromosome changes are often found in the bone marrow (BM) of patients with SDS. Among them, the most frequently observed clonal abnormalities are an isochromosome of the long arm of chromosome 7, i(7)(q10), and an interstitial deletion of the long arm of chromosome 20, del(20)(q) [9]. We have already shown those molecular mechanisms underlying the fact that both these more frequent anomalies are benign prognostic signs, as they are not associated with leukemia progression. The i(7)(q10) results in duplication of mutation c.258 + 2 T > C of the *SBDS* gene, which is a hypomorphic mutation that allows the production of a scant amount of functional protein [10]. The del(20)(q) results in deletion of the *EIF6* gene [11, 12]. Both these anomalies are predicted to result in more efficient ribosome biogenesis in the BM abnormal clone which may lower the risk of MDS/AML [10, 11] and result in a milder haematological condition compared to SDS patients with other chromosome changes or with normal karyotype [13–15]. These conclusions were further supported by a recent paper concerning the benign effects of somatic mutations of *EIF6* [16].

We have demonstrated that SDS is not associated with an increase of spontaneous chromosome breaks, as in customary breakage syndromes, a possibility that was raised by some previously published reports [17]. Nevertheless, we suggested since 2000 that SDS may be associated with a particular type of karyotype instability resulting in specific anomalies of chromosomes 7 and 20: i(7)(q10) and del(20)(q) [18].

We present here an updated overview of available cases with del(20)(q) from a cohort of patients in whom the deletion was defined precisely by array-based comparative genomic hybridization (a-CGH) and fluorescence in situ hybridization (FISH). These findings support the notion that the selection pressures exerted by *SBDS* deficiency result in specific karyotype instability (confirmed by some novel data presented here) and recurrent clonal abnormalities.

## Results

**Fig. 1:**
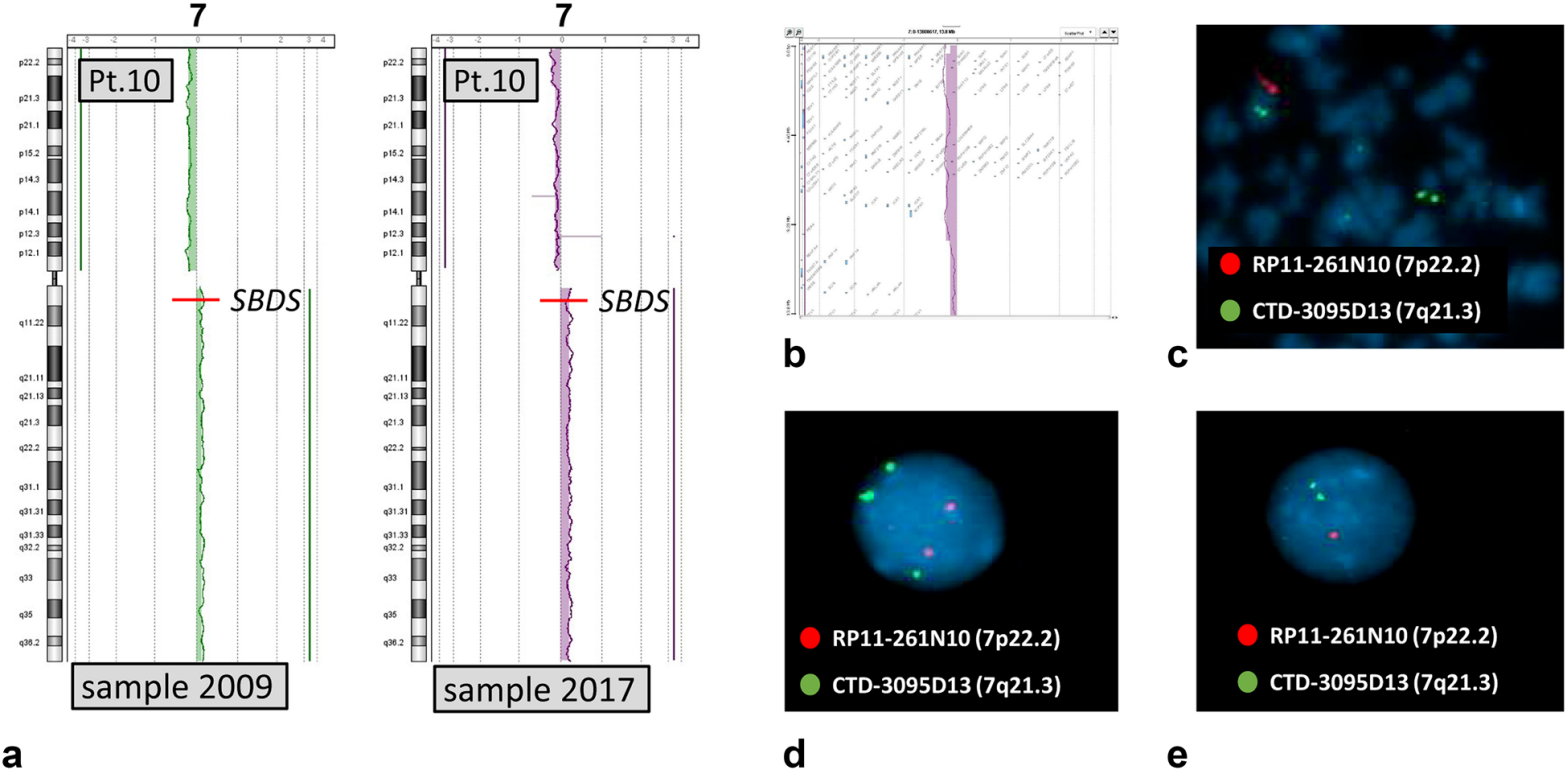
Profiles of a-CGH of patient 10 in the BM samples drawn in 2009, showing the i(7)(q10), and in 2017 also showing a deletion of the telomeric region of the short arm (**a**), which is more appreciable in an enhanced image (**b**). Double color FISH with the probes indicated in the figure (one located in the short arm, the other in the long arm) shows that two different abnormal clones are present: one with the i(7)(q10) (nucleus with three green and two red signals) (**d**), one without the i(7)(q10) and with the deletion as demonstrated by the mitosis with one normal chromosome 7 and a deleted one, with only the green signal (**c**), and by the nucleus with two green and one red signal (**e**)

The only patient considered here with the i(7)(q10), patient 10, later acquired also a deletion of the short arm of chromosome 7 (Table 1, Fig. 1), which was demonstrated by FISH as present in a clone without the isochromosome. Figure 1a shows the a-CGH profile of the i(7)(q10) in BM samples obtained in 2009 and 2017. An enlarged view of the short arm telomeric region from the sample obtained in 2017 demonstrates the deleted segment (Fig. 1b). FISH analysis demonstrates that the clones with the i(7)(q10) and with the del(7)(p21.3pter) are independent (Fig. 1c, d and e).

**Table 1:** Patients with more than one clonal anomaly in BM, either in the same sample or in samples drawn in different years

Patient	Sample	Anomaly 1st clone—bands and DNA bp (% cells)	Anomaly 2nd clone—bands and DNA bp (% cells)
2	2009	del(20)(q11.21q13.13) 30,733,183–49,339,757 bp (43%)	del(20)(q11.22q11.23) 33,148,327–36,779,644 bp (23%)^a^
4	2010	del(20)(q11.21q13.13) 31,954,597–49,216,901 bp (17%)^b^ dup(20)(q13.32–q13.33) 58,349,436–60,320,956 bp (19%)^b^	dup(3)(q24q29) 143,044,212–195,076,511 bp (34%)^b^
9	2009	del(20)(q11.21q13.32) 30,876,455–57,739,561 bp (55%)	–
	2017	–	del(20)(q11.21q13.13) 30,904,022–49,344,382 bp (52%)
10	2009	i(7)(q10) (24%)^c^	–
	2017	i(7)(q10) (16%)^c,d^	del(7)(p21.3pter) (10%)^d^
13	2018	del(20)(q11.22q11.23) 32,738,995–34,468,385 bp (35%)	del(20)(q11.2q13)^e^ 2 out of 6 mitoses
14	2017	del(20)(q11.21q12) 31,814,242–40,237,993 bp (12%)^b^ dup(20)(q11.33) 58,725,726–61,314,465 bp (18%)^b^	–
	2019	del(20)(q)^f^	del(20)(q)^f^

Chromosomal anomalies were defined by a-CGH except where noted otherwise. The percentage of abnormal cells is calculated from a-CGH results, except for the 2^nd^ clone of patient 13 and the 2019 sample of patient 14

^a^This percentage refers only to the 2nd clone ^b^ The anomalies may be in independent clones or associated in a single clone

^c^a-CGH results showed monosomy of the entire short arm and trisomy of the entire long arm

^d^The anomalies were in two independent clones, as shown by FISH (Fig. 1c, d, e)

^e^Result of chromosome analysis

^f^Two clones with different del(20)(q), as shown by double color FISH with two different probes identifying different regions of chromosome 20 long arm (Fig. 3 D)

**Fig. 2:**
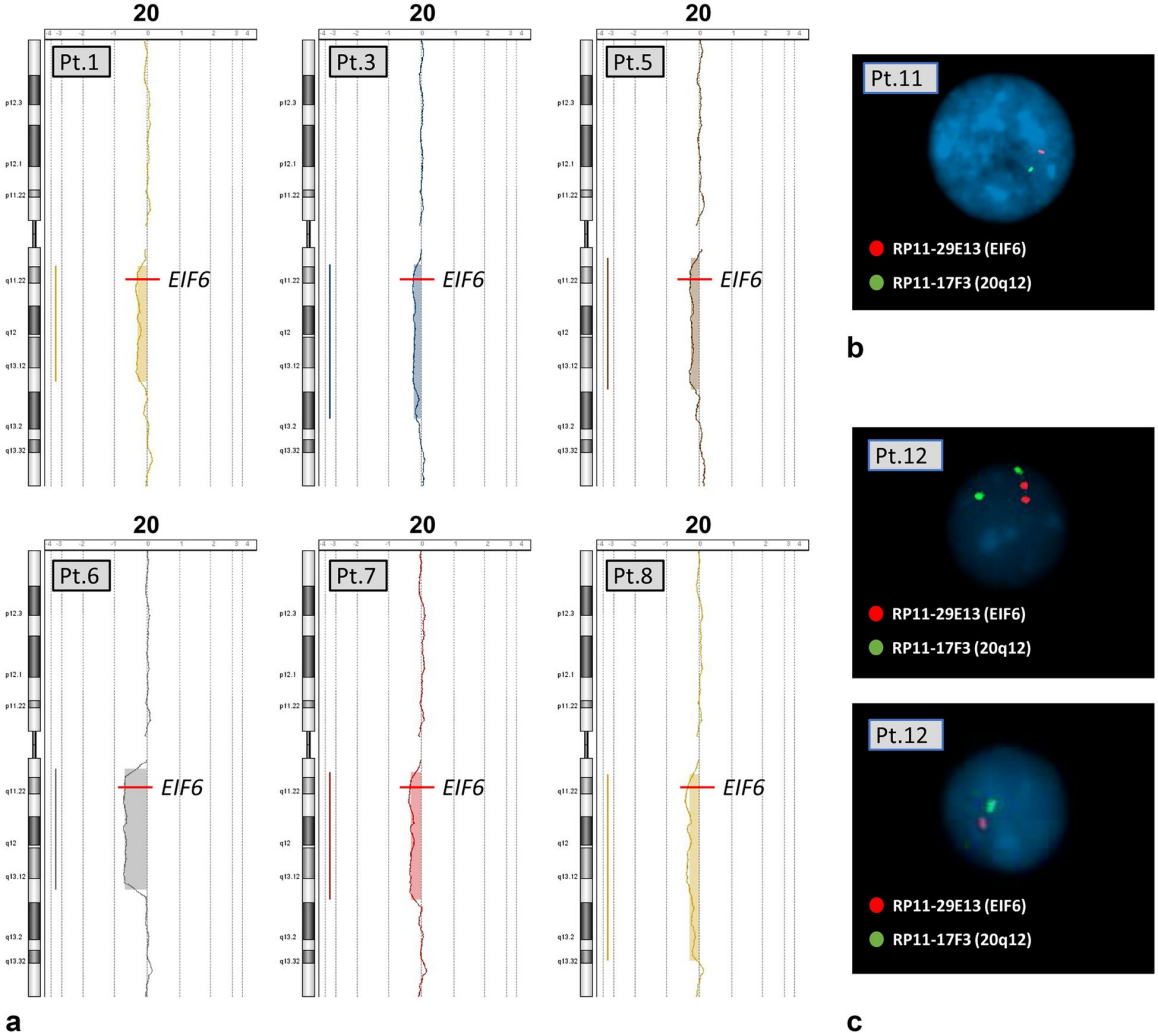
Patients with del(20)(q) as sole anomaly: a-CGH profiles of six patients (**a**), and FISH results on nuclei with probes RP11-29E13 (red signal), which includes the *EIF6* gene, and RP11-17F3 (green), localized in band 20q12. Patient 11, with one single signal of both probes (**b**), indicating a large deletion; Patient 12, with one normal nucleus with two signals of both probes and one nucleus with one signal for both probes (large deletion) (**c**). The precise definition of the deletions is in Table 2

The del(20)(q) was defined precisely by a-CGH and FISH in the 13 patients in whom it was initially noted on chromosomal karyotype analysis. Eight patients manifested the del(20)(q) as their sole chromosomal anomaly. The positions of the deletion breakpoints, together with the proportion of cells harboring the deletion are listed in Table 2. The a-CGH profiles of six of these patients are shown in Fig. 2a. Table 2 includes also the two cases (11 and 12) in whom the paucity of BM abnormal cells did not allow the a-CGH study, and the result derives only from FISH on interphase nuclei (Fig. 2b and c). In particular, we observed 18/321 (5.6%) and 9/309 (2.9%) nuclei with the deletion of chromosome 20 in patient 11 and 12, respectively. FISH on nuclei of normal healthy control gave 100% of normal signals for both probes as control. It is possible that the FISH results in these two patients could also indicate a clone with monosomy 20, but we interpreted our findings as due to an interstitial deletion because during standard chromosome analyses across years, we always found a clone with the interstitial deletion of chromosome 20, del(20)(q), but never a monosomy 20. The dimension of the deletion of these two patients is at least of about 7 Mb according to the segment spanning the two probes used (Table 2),

**Table 2:** Patients with del(20)(q) as sole anomaly in BM: definition of the deletion by a-CGH (patients 1–8) or FISH (patients 11 and 12)

Patient	Bands	Deletion start (bp)	Deletion stop (bp)	% Abnormal cells
1	q11.21-q13.13	31,954,597	48,328,296	36%
3	q11.2-q13.2	31,720,622	53,559,811	26%
5	q11.21-q13.13	30,849,566	49,398,586	29%
6	q11.21-q13.13	30,889,915	47,912,299	74%
7	q11.21-q13.13	31,412,080	49,339,757	39%
8	q11.21-q13.32	31,671,222	57,911,624	36%
11	q11.22-q12^a^	–	–	5.6%^b^
12	q11.22-q12^a^	–	–	2.9%^c^

The percentage of abnormal cells is calculated from a-CGH results, except for patients 11 and 12

^a^Result obtained by double color FISH with the bac probes RP11-17F3 and RP11-29E13: the deletion therefore is at least from 33,797,020 to 40,857,566 bp

^b^% from FISH results: 1/16 mitoses and 18/321 nuclei

^c^% from FISH result: 9/309 nuclei

Five patients with the del(20)(q), showed more than one abnormal clone, either simultaneously in a single BM sample (cases 2, 4, and 13), or sequentially in two samples drawn in different years (cases 9 and 14). The results concerning these patients are summarized in Table 1. The percentage of abnormal cells in Tables 1 and 2 was calculated from a-CGH data as previously described [19].

**Fig. 3:**
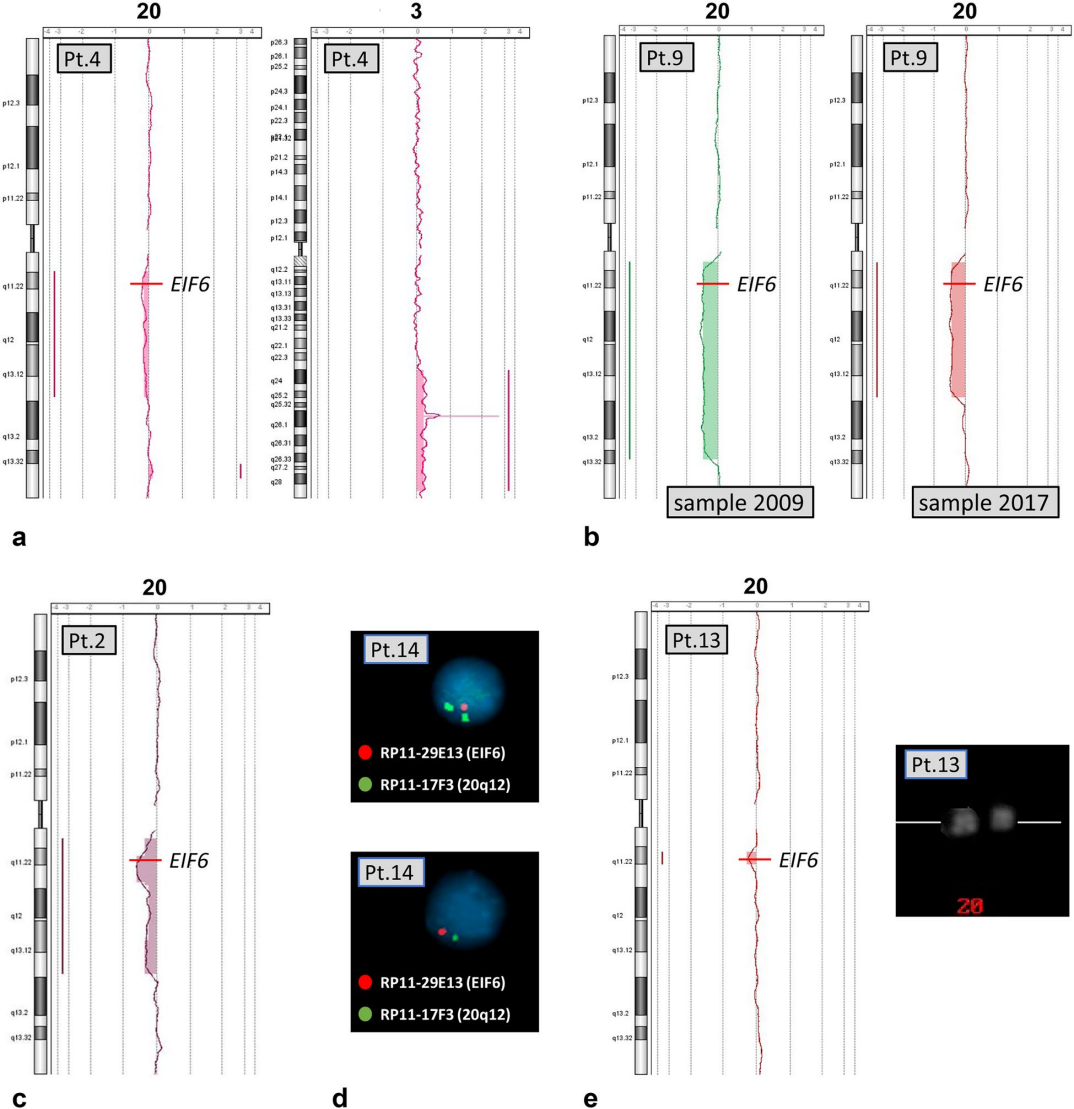
Profiles of a-CGH and FISH signals in patients with more than one abnormal clone. The a-CGH profiles of chromosome 20 and chromosome 3 of patient 4 show a large deletion del(20)(q), a small duplication of the long arm of chromosome 20 and a large duplication of chromosome 3 (**a**). The a-CGH profiles of BM samples from patient 9 drawn in 2009 and 2017 show two distinct sequential clones with deletions of different sizes (**b**). The a-CGH profile of patient 2 shows two overlapping deletions in (20)(q), one larger and one smaller, included in the first one, and both leading the loss of the *EIF6* gene (**c**). The double color FISH on nuclei of patient 14 at the BM sample drawn in 2019 (**d**), with two different probes (indicated in the figure), demonstrates that one clone bear a large deletion (loss of both signals, below), and another clone a smaller deletion, losing only the region of the *EIF6* gene (only one red signal, above). The a-CGH profile of patient 13 with a tiny del(20)(q), and a cut-out of Q-banded chromosomes 20 in which the deletion, although small, is large enough to be detected (**e**). The precise delineation of the anomalies is summarized in Table 1

The results for each patient with more than one abnormal clone were further analyzed. The a-CGH profile of patient 2 (Table 1, Fig. 3c) showed in the same BM sample not only a large deletion in 43% of the cells but also a second clone with a smaller deletion, inside the region of the first one, in 23% of the cells. The overall percentage of abnormal cells became 66% for this smaller segment. Patient 4 showed a large deletion del(20)(q), a small duplication of 1.9 Mb, dup(20)(q13.32q13.33), and a duplication of the long arm of chromosome 3 (Table 1, Fig. 3a): these anomalies, found in the same BM sample, might be present in the same clone or in independent clones. In patient 9, the deletion was defined by a-CGH in 2009, but it became shorter in 2017 (Table 1, Fig. 3b), consistent with the development of two independent clones each with different deletions, possibly at different times. Similarly, two clones with different deletions of chromosome 20 were identified in patient 13 by comparing the results of standard chromosome analyses and a-CGH performed in 2018 (Table 1). A large del(20)(q) was observed by chromosome analysis (it was already identified one year earlier), however, a-CGH showed only a very small deletion (the smallest we ever found, 1.7 Mb (Table 1, Fig. 3e)). These findings are consistent with the presence of two clones with two different deletions, as the clone observed by a-CGH was too small to be identified through chromosome analysis, despite its presence in 35% of the cells. On the contrary, the large deletion identified at standard chromosome analysis should be detectable by a-CGH, but it must be present in a small clone, as it escaped identification by this method.

In patient 14, the del(20)(q) was found in 2017, and defined by a-CGH, which also gave evidence of a small duplication of chromosome 20 of 2.8 Mb, dup(20)(q11.33) (Table 1). In 2019, standard chromosome analysis failed to show the deletion, but FISH on nuclei with two different probes demonstrated the presence of two different deleted clones (Table 1, Fig. 3d), as 4% (9/222 nuclei) of the cells lost both the signals of the probes used, while 6% (13/222 nuclei) lost only the one of the probe including the *EIF6* gene (Control FISH on normal donor nuclei gave a 100% of normal signals). Every del(20)(q) clonal abnormality resulted in a deletion of the *EIF6* gene.

## Discussion

This study provides a detailed analysis of an additional 13 SDS patients with del(20)(q) which, taken together with the patients previously reported, demonstrate that *EIF6* is lost in all del(20)(q) clones observed in a total of 24/24 patients analyzed until now. Overall, among 22 of them, the result was reached by means of a-CGH, and, in two cases, through FISH analysis with informative probes (Table 2, Additional File 1: Table S1). It has been postulated that del(20)(q), with consistent loss of the *EIF6* gene in all cases analysed, imply a good prognosis, with lower risk of MDS/AML and milder haematological conditions [11, 13, 15]. The relevance of EIF6 protein in ribosome biogenesis [4] may explain the benign effects in BM of the deletion del(20)(q) [16]. In a study on gene expression in BM of SDS patients with del(20)(q), with i(7)(q10), with other clonal anomalies or with normal karyotype, the transcription pattern of the patients with acquired del(20)(q) was similar to that of healthy subjects, at least in cases with a high proportion of abnormal cells, thus supporting the potential positive role of this anomaly [20].

Considering all the 24 patients analyzed in the present paper or previously reported, and belonging to two large different cohorts, our results confirmed that all the deletions are interstitial, with proximal breakpoints clustered in a region of about 2.6 Mb in bands q11.21-q11.22, while distal breakpoints were variable (Tables 1 and 2, Additional File 1: Table S1). In the majority of the cases, the deletion was large enough to be identified by standard chromosome analysis (range 14–26.9 Mb in 18 out of 24 patients), though smaller deletions were detectable only by a-CGH or FISH in a minority of cases. Overall, the range of the losses varies from 1.7 Mb (patient 13) to 26.9 Mb (patient 9) (Table 1, Additional File Table S1).

The six patients who showed unexpected clonal variations of the anomalies in BM, del(20)(q) in five cases, i(7)(q10) in one, either in a single sample, or in samples obtained in different years (Table 1) deserve a specific comment. We have to remark that, besides these six cases, further evidence of peculiar instability is offered by three patients already reported (UPN 17, 20 and 68) [12], in whom the del(20)(q) showed a further rearrangement leading to complex independent subclones (UPN 17 and 20) or two separate deletions with a segment conserved between (UPN 68). The molecular basis underlying this mechanism have not been clarified yet. A possible hypothesis is that the karyotype instability, typical of SDS and demonstrated by our findings, may drive to different *scenarios*: 1) Karyotype instability driving to rearrangements of chromosomes 7 or 20, could have a positive effect for the clone that carries the anomaly; this could be related to the doubling of the hypomorphic *SBDS*^*258*+*2 T*>*C*^ mutation on the i(7)(q10) [10] or by deletion of one *EIF6* allele in the del(20)(q) [13], respectively. 2) Karyotype instability driving to rearrangements of other chromosomes, could be of unknow significance or even have potentially negative effects. 3) The karyotype instability could continuously act in SDS patients and drive to different independent rearrangements in patients during life span. These latter successive rearrangements can further involve chromosomes 7 and 20 (with possible benign effects as above) or other chromosomes (with unknown significance). The data regarding the six new cases presented here, strongly confirm that in the BM of SDS patients the karyotype instability is actually present and acts mainly on chromosomes 7 and 20, being the basis of the recurrent clonal anomalies, through a striking selective pressure.

## Conclusions

The present paper leads to conclusions concerning two points:

We confirm that in all patients with SDS, the del(20)(q) in BM imply the loss of the *EIF6* gene: the analysis of the cases presented here and of those already reported leads to a total of 24 patients. This change has specific prognostic benign implications, playing a role as BM rescue mechanisms as previously postulated [13, 15, 16, 20].

The patients who bear the i(7)(q10) or the del(20)(q) in BM, may rearrange the abnormal clones in the course of the disease, or they may loose and then reconstruct de novo the deletion, or they may evolve independently these clones in other ways. In particular, in 4/6 cases with del(20)(q) and unexpected clonal evolution here reported, the variated parallel clones involve invariably the chromosome 20, again with the loss of *EIF6*. In 1/6 cases, the patient with the i(7)(q) rearranged independently the normal chromosome 7 in a different clone. These findings strongly indicate that in SDS patients there is a peculiar karyotype instability, depending upon a mechanism/s not yet identified, that, through a striking and specific selective pressure, acts mainly on chromosomes 7 and 20 and drives to a somatic rescue of the clone, as recently shown by Tan and coworkers for del(20)(q) [16].

## Methods

Bone marrow samples were obtained from eight patients from the cohort of the North American Shwachman-Diamond Syndrome Registry (SDSR) and from six patients from the cohort of the Registro Italiano per la Sindrome di Shwachman-Diamond (RI-SDS). In total, samples were analyzed from fourteen patients, three females and eleven males, with age at the time of sampling for the analyses reported here ranging from three to 29 years (Table 3). All the patients presented the main typical phenotypic signs of SDS. Informed consent for this study was obtained according to the principles of the Declaration of Helsinki from the patients or the patients’ parents on protocols approved by the institutional review boards for human subjects research.

**Table 3:** Patient characteristics

Patient ID ^a^	Age^b^/sex	*SBDS* mutations
1	6/M	c.183_184delTAinsCT/c.258 + 2 T > C
2	4/M	c.258 + 2 T > C/c.258 + 2 T > C
3	20/F	c.183_184delTAinsCT/c.258 + 2 T > C
4	5/M	c.183_184delTAinsCT/c.258 + 2 T > C
5	11/M	c.170 T > C/258 + 2 T > C
6	14/M	c.258 + 2 T > C/c.258 + 2 T > C
7	29/M	c.183_184delTAinsCT/c.258 + 2 T > C
8	16/M	c.183_184delTAinsCT/c.258 + 2 T > C
9	11, 19/M	c.183_184delTAinsCT + 258 + 2 T > C/258 + 2 T > C
10	13, 16/F	c.183_184delTAinsCT/c.258 + 2 T > C
11	15/F	c.183_184delTAinsCT/c.258 + 2 T > C
12	M	c.183_184delTAinsCT/c.258 + 2 T > C
13	13, 14/M	c.183_184delTAinsCT + 258 + 2 T > C/c.258 + 2 T > C
14	3, 5/M	c.183_184delTAinsCT + 258 + 2 T > C/c.258 + 2 T > C

^a^Patients 1–8 from the cohort of the North American Shwachman-Diamond Syndrome Registry (SDSR); patients 9–14 from the cohort of the Registro Italiano per la Sindrome di Shwachman-Diamond (RI-SDS)

^b^Age (years) at time or times of BM sampling

Mutational analysis was performed according to Boocock et al. [1] or by sequencing the entire *SBDS* gene, and the mutations of patients are indicated in Table 3. These patients were chosen because they bore the more frequent clonal chromosome anomalies in BM, the del(20)(q) in thirteen patients and the i(7)(q10) in one, which were detected by chromosome analysis.

Chromosome analyses were performed on BM by QFQ-banding with routine methods. Karyotypes was reconstructed by Leica Chantal Software. FISH on BM mitoses and nuclei was carried out according to standard techniques with the BAC probes informative for the change detected in each patient: dual color FISH with probes RP11-17F3 and RP11-29E13 (Empire Genomics, Williamsville, New York, USA) for patients 9, 11, 12, 14 and dual color FISH with probes RP11-261N10 and CTD-3095D13 (Empire Genomics, Williamsville, New York, USA) for Patient 10). The two dual color FISH was tested on peripheral blood nuclei from a healthy control subject to assess the threshold for the detection of the deletion: every probe used, gave 300/300 (100%) normal signals in the hybridized nuclei.

The a-CGH was performed on DNA from BM samples with the 4 × 180 K or 244 K genome-wide system (Agilent Technologies Inc., Santa Clara, CA, USA), according to the manufacturer’s instructions, as already described [21]. Briefly, DNA from BM was extracted by Qiagen Flexigene kit (Qiagen, Hilden, Germany). The normal reference DNA and the patient’s DNA were labelled with Cy5 and Cy3 fluorochromes respectively and purified by the use of SureTag Complete DNA Labeling Kit (Agilent Technologies Inc., Santa Clara, CA, USA). Labelled DNAs were dissolved into the Oligo aCGH/ChIP-on-chip Hybridization solution (Agilent Technologies Inc., Santa Clara, CA, USA) in a suitable volume for hybridization, according to manufacturer’s instructions. Hybridization was performed in a rotating oven at 65 °C for 24 h (180 K slides) or 40 h (240 k slides). Microarray slides were washed with Agilent’s Oligo aCGH/ChIP-on-chip solution 1 and 2 according to manufacturer’s instructions and slides images were acquired with Agilent’s G2565CA microarray scanner. Features were extracted by Agilent’s Feature Extraction 12.1.1.1 software and data analysis were performed by Agilent’s Genomic Workbench 7.0.4.0 software. All map positions in the results refer to the genome assembly map hg19.

Chromosome, FISH or aCGH analyses were repeated in all patients during several years, at least once per year. Results of chromosome analysis and a-CGH of patients 9, 10, and 11 have been previously partially reported [13, 17, 22].

## Supplementary Information

**Additional file 1:Supplemental Table S1**. The del(20)(q) start/stop breakpoints (obtained by a-CGH or FISH) and the extent of the del(20)(q) are summarized for all 25 patients carrying the del(20)(q) in the present report and prior publications. Patients 2 and UPN68 carry two separate del(20)(q) deletions as indicated by the breakpoints in the table.

## Data Availability

The data used and analyzed in the current study are available from the corresponding author on reasonable request.

## Abbreviations

SDS: Shwachman-Diamond syndrome
MDS: Myelodysplastic syndrome
AML: Acute myeloid leukaemia
BM: Bone marrow
a-CGH: Array-based comparative genomic hybridization
FISH: Fluorescent in situ hybridization
SDSR: North American Shwachman-Diamond Syndrome Registry
RI-SDS: Registro Italiano per la Sindrome di Shwachman-Diamond
UPN: Unique patient number
