# Supplementary material for: The frequent and clinically benign anomalies of chromosomes 7 and 20 in Shwachman-diamond syndrome may be subject to further clonal variations
Source: Mol Cytogenet. 2021 Nov 24;14:54. doi: 10.1186/s13039-021-00575-w (PMC8611838; doi:10.1186/s13039-021-00575-w)
Supplement: Supplementary file 1 — Additional file 1:Supplemental Table S1. The del(20)(q) start/stop breakpoints (obtained by a-CGH or FISH) and the extent of the del(20)(q) are summarized for all 25 patients carrying the del(20)(q) in the present report and prior publications. Patients 2 and UPN68 carry two separate del(20)(q) deletions as indicated by the breakpoints in the table. [file 13039_2021_575_MOESM1_ESM.docx]

**Supporting Informations**

**Supplemental Table S1:** The del(20)(q) start/stop breakpoints (obtained by a-CGH or FISH) and the extent of the del(20)(q) are summarized for all 25 patients carrying the del(20)(q) in the present report and prior publications. Patients 2 and UPN68 carry two separate del(20)(q) deletions as indicated by the breakpoints in the table.

| Reference | Patient ID | Specimen | Deletion Start | Deletion stop | Extent (bp) |
| --- | --- | --- | --- | --- | --- |
| Present report | 1 | 2018 | 31954597 | 48328296 | 16,373,699 |
| Present report | 2 | 2009 | 30733183  33148327 | 49339757  36779644 | 18,606,574  3,631,317 |
| Present report | 3 | 2018 | 31720622 | 53559811 | 21,839,189 |
| Present report | 4 | 2010 | 31954597 | 49216901 | 17,262,304 |
| Present report | 5 | 2016 | 30849566 | 49398586 | 18,549,020 |
| Present report | 6 | 2011 | 30889915 | 47912299 | 17,022,384 |
| Present report | 7 | 2018 | 31412080 | 49339757 | 17,927,677 |
| Present report | 8 | 2017 | 31671222 | 57911624 | 26,240,402 |
| Present report | 9 | 2009 | 30876455 | 57739561 | 26,863,106 |
|  |  | 2017 | 30904022 | 49344382 | 18,440,360 |
| Present report | 11 | 2019 | 33797020* | 40857566* | 7,060,546** |
| Present report | 12 | 2019 | 33797020* | 40857566* | 7,060,546** |
| Present report | 13 | 2017 | 32738995 | 34468385 | 1,729,390 |
| Present report | 14 | 2017 | 31814242 | 40237993 | 8,423,751 |
| Valli et al, 2013^9^ | UPN 14 | 2004 | 31163090 | 35309353 | 4,146,263 |
| Valli et al, 2013^9^ | UPN 17 | 2008 | 31205853 | 55894832 | 24,688,979‬ |
| Valli et al, 2013^9^ | UPN 20 | 2004 | 31294381 | 57252304 | 25.957.923 |
| Valli et al, 2013^9^ | UPN 65 | 2012 | 30157286 | 49497910 | 19.340.624‬ |
| Valli et al, 2013^9^ | UPN 68 | 2013 | 31262228  45244728 | 43141564  47373129 | 11.879.336  2.128.401‬ |
| Valli et al, 2019^10^ | UPN 1 | 2001 | 31891819 | 48287277 | 16.395.458 |
| Valli et al, 2019^10^ | UPN 6 | 2006 | 30922628 | 49497969 | 18.575.341‬ |
| Valli et al, 2019^10^ | UPN 35 | 2016 | 31798183 | 47884947 | 16.086.764 |
| Valli et al, 2019^10^ | UPN 82 | 2014 | 30020250 | 52206444 | 22.186.194 |
| Valli et al, 2019^10^ | UPN 84 | 2016 | 32620650 | 58600338 | 25.979.688‬ |
| Valli et al, 2019^10^ | UPN 85 | 2015 | 31814242 | 36538658 | 4,724,416‬ |

* From FISH: approximate minimal evaluation

** minimal evaluation
